# Gut Microbiota Biomarkers in Patients with Hepatocellular Carcinoma in the Era of Immune Checkpoint Inhibitors

**DOI:** 10.3390/life16040641

**Published:** 2026-04-10

**Authors:** Maria Cerreto, Marta Maestri, Maria Pallozzi, Lucia Cerrito, Leonardo Stella, Gianluca Ianiro, Antonio Gasbarrini, Francesca Romana Ponziani

**Affiliations:** 1 Liver Unit—Centro Malattie dell’Apparato Digerente (CEMAD), Medicina Interna e Gastroenterologia, Fondazione Policlinico Universitario Gemelli IRCCS, 00168 Rome, Italy; mariacerreto@gmail.com (M.C.); marta.maestri1@guest.policlinicogemelli.it (M.M.); maria.pallozzi@guest.policlinicogemelli.it (M.P.); lucia.cerrito@policlinicogemelli.it (L.C.); leonardo.stella@guest.policlinicogemelli.it (L.S.); gianluca.ianiro@policlinicogemelli.it (G.I.); antonio.gasbarrini@unicatt.it (A.G.); 2 Dipartimento di Medicina e Chirurgia Traslazionale, Università Cattolica del Sacro Cuore, 00168 Rome, Italy

**Keywords:** immune checkpoint inhibitors, hepatocellular carcinoma, gut microbiota, metabolites

## Abstract

Immune checkpoint inhibitors (ICIs) have revolutionized the therapeutic landscape for hepatocellular carcinoma (HCC); however, a considerable proportion of patients do not achieve durable clinical benefits. This highlights the need for reliable predictive biomarkers, which are currently lacking. The accumulated evidence supports a relevant role of the gut–liver axis in modulating immunotherapy outcomes, and several studies have identified distinct microbial features associated with either responders or non-responders. Responders to immunotherapy frequently present with higher microbial diversity and enrichment of beneficial taxa, whereas the expansion of pro-inflammatory and pathogenic bacteria has been associated with primary resistance and increased treatment-related toxicity in non-responders. However, the available findings remain heterogeneous across cohorts, likely owing to differences in geography, diet, liver disease etiology, treatment regimens, and microbiome analytical methods. Machine-learning models integrating metagenomic and metabolomic data have shown encouraging results in defining microbial signatures associated with treatment outcomes, although variability among cohorts currently limits their clinical applicability and generalizability. Beyond microbial taxonomic composition, microbiota-derived metabolites—such as short-chain fatty acids, bile acids, inosine, and tryptophan catabolites—appear to play a crucial role in shaping the tumor microenvironment and host immune responses, thus representing additional candidate biomarkers, also due to the relative ease of their measurement. Finally, microbiota-targeted interventions are emerging as potential strategies to enhance immunotherapy efficacy. Overall, the gut microbiome and its metabolic activity represent promising tools, albeit still under investigation, for patient stratification and personalized management in HCC treated with ICIs. Therefore, this review aims to summarize and critically discuss the current evidence on gut microbiota-derived biomarkers of response and resistance to ICIs in HCC, with particular focus on microbial composition, microbiota-related metabolites, and emerging microbiome-based therapeutic strategies. This narrative review provides an updated overview of the role of gut microbiota as both a biomarker and a therapeutic target in patients with hepatocellular carcinoma (HCC) receiving immune checkpoint inhibitor (ICI) therapy.

## 1. Introduction

Immunotherapy, whose efficacy has been extensively demonstrated in malignancies such as melanoma and non-small-cell lung cancer, has now become one the cornerstones in the management of HCC. In particular, increasing attention has been directed toward ICIs, including cytotoxic T-lymphocyte antigen 4 (CTLA-4) and programmed death-1 (PD-1)/programmed death-ligand 1 (PD-L1) inhibitors, for their ability to enhance anti-tumor immune responses by restoring T-cell activity and preventing the immune escape mechanisms adopted by tumor cells [1,2,3].

Currently, the approved first-line systemic treatment for hepatocellular carcinoma is the combination of Atezolizumab (anti-PD-L1)–Bevacizumab (anti-VEGF) [4,5]. More recently, the HIMALAYA trial demonstrated a significant improvement in overall survival (OS) with the combination of durvalumab (anti-PD-L1) and tremelimumab (anti-CTLA-4), leading to its FDA approval as an alternative first-line regimen [6]. Additional ICIs, such as pembrolizumab and nivolumab (both anti-PD-1) and ipilimumab (anti-CTLA-4), have been approved by the FDA for second-line treatment of HCC.

Despite these advances, nearly 60–70% of patients fail to respond to ICI therapy due to the development of resistance [7]. In melanoma, around 60% of patients show primary resistance and 20–30% develop acquired resistance after an initial responsive phase in both the adjuvant setting and the advanced setting [8]; in non-small-cell lung cancer (NSCLC), resistance rates settled around 30–40%, especially in PD-L1 inhibitors [9]. In HCC, approximately 70 to 80% of patients develop resistance to ICIs, showing a significantly lower response rate to these therapies compared to other solid tumors [10]. One of the main challenges is the absence of reliable serological or tissue biomarkers capable of identifying patients most likely to benefit from immunotherapy and those at risk of primary or acquired resistance [11]. Currently, the only FDA-approved predictive biomarkers for ICI use across malignancies are PD-L1 expression, microsatellite instability/deficient DNA mismatch repair (MSI/dMMR) and tumor mutational burden (TMB) [12].

In this challenging scenario, the integration of innovative technologies such as genomics, transcriptomics, metabolomics and microbiomics is opening new perspectives for biomarker discovery. In particular, gut microbiota profiling has emerged as a promising, non-invasive tool to predict an individual patient’s likelihood of responding to immunotherapy [13].

## 2. Interactions Between Gut Microbiota and ICIs

It is well established that the gut microbiota composition and, in particular, the condition of gut dysbiosis play a relevant role in a number of infectious and auto-immune diseases. Moreover, recent evidence shows that the gut microbiota and microbiome-derived metabolites also have a critical role in carcinogenesis and, conversely, in the response to chemotherapic and immunotherapic treatments, by shaping the immune system’s capacity to recognize and eliminate tumor cells [11,14,15]. This section will move from biological mechanisms, to clinical association studies, to predictive modeling and clinical implications.

### 2.1. Biological Rationale for Microbiome–ICI Interactions

Mechanistically, the gut microbiota modulates ICI efficacy through several pathways. Dendritic cells sense microbial antigens and present them to T cells in lymph nodes, inducing central memory T cells (TCMs) and their differentiation into effector T cells. This process increases production of interleukin-2 (IL-2) and interferon-γ (IFN-γ), both of which potentiate ICI activity by enhancing antigen presentation, boosting natural killer (NK) cell cytotoxicity and promoting lysosomal activity in macrophages [16]. For example, in responders *A. muciniphila* stimulates IL-12 secretion by dendritic cells, recruiting memory CD4^+^ T cells and enhancing ICI responses [17]. Similarly, *B. fragilis* and other taxa from the genera *Bacteroides*, *Ruminococcaceae*, *Eubacterium and Fusobacterium* activate IFN-γ-producing CD8^+^ T cells. Conversely, dysbiotic microbiota in non-responders promote expansion of IL-10-producing regulatory T cells, supporting tumor progression [18].

### 2.2. Clinical Evidence Linking Microbial Profiles to ICI Response

These immunological mechanisms provide the biological basis for the clinical observation that distinct microbial profiles are associated with differential response to ICIs in HCC. Consistent with this hypothesis, several studies have reported significant differences in microbial α-diversity and composition between responders and non-responders to ICIs. Specific bacterial taxa—including *Faecalibacterium*, *Bifidobacterium*, *Lachnospiraceae*, *Akkermansia* spp., *Ruminococcaceae* and *Prevotellaceae*—have repeatedly emerged as potential predictors of treatment response [19]. For instance, in a study by Routy et al. involving patients with non-small-cell lung cancer (NSCLC) and renal cell carcinoma (RCC) treated with PD-1 inhibitors, *Akkermansia muciniphila*, *Enterococcus faecium* and *Alistipes* indistinctus were more abundant in responders compared with non-responders [17]. Similarly, in a pilot study by Sarkar et al., pre- and on-treatment gut microbiota of NSCLC patients receiving pembrolizumab (anti-PD-1) were compared. While baseline α-diversity did not differ between groups, post-treatment responders exhibited a significant enrichment in *Clostridium sensu stricto 1* and a reduction in *Odoribacter*, *Gordonibacter*, *Candidatus Stoquefichus*, *Escherichia-Shigella* and *Collinsella*. Conversely, non-responders displayed an increase in *Prevotella*, *Porphyromonas*, *Streptococcus* and *Escherichia-Shigella*, along with a marked decrease in *Akkermansia* [20].

In gastrointestinal cancers, Peng et al. observed that a higher *Prevotella/Bacteroides* ratio correlated with improved responses to anti-PD-1/PD-L1 therapy in advanced disease [21]. The DELIVE clinical trial in advanced gastric cancer identified *Veillonella* abundance as being associated with disease remission or stabilization, suggesting its potential as a tumor-specific biomarker, although results across studies remain inconsistent [22]. For example, Gao et al. reported that elevated *Fusobacterium nucleatum* levels were linked to higher response rates and longer progression-free survival (PFS) in colorectal cancer (CRC) patients treated with PD-1 inhibitors, possibly via recruitment of IFN-γ-producing CD8^+^ tumor-infiltrating lymphocytes (TILs) [23]. In contrast, Jiang et al. found that poor responders with metastatic CRC had increased *F. nucleatum* and its metabolite succinic acid, which may suppress the cGAS–interferon-β pathway, thereby impairing CD8^+^ T-cell trafficking to the tumor microenvironment. Supporting this mechanism, metronidazole treatment reduced *F. nucleatum* abundance and succinate levels, enhancing ICI efficacy [24].

In melanoma, responders to PD-1/CTLA-4 inhibitors often display enrichment in *Bifidobacterium longum*, *Collinsella aerofaciens* and *E. faecium* [25], with similar findings replicated in multiple cohorts, including consistent associations between *A. muciniphila* and improved ICI response [26,27]. While most investigations have focused on PD-1/PD-L1 blockade, the gut microbiome also influences responses to CTLA-4 inhibition. Notably, anti-CTLA-4 efficacy has been shown to require specific *Bacteroides* species: resistance in germ-free or antibiotic-treated mice was overcome by administration of *Bacteroides fragilis* or *B. fragilis*-specific T cells [28].

A 2023 systematic review examined the associations between ICI responses (anti-PD-1, anti-PD-L1, anti-CTLA-4) and gut microbiota composition in patients with solid tumors [29]. The most distinctive taxa in responders included *Faecalibacterium prausnitzii*, *Streptococcus parasanguinis*, *Bacteroides caccae* and *Prevotella copri*, while *Bacteroides obeum* and *B. ovatus* were characteristic of non-responders. Particularly, *F. prausnitzii*—an anaerobe reduced in many intestinal disorders—appears to maintain intestinal homeostasis and reduce inflammation, thereby limiting tumor-promoting effects [30]. Supporting this, a study in 26 melanoma patients treated with ipilimumab (CTLA-4 inhibitor) found that baseline enrichment in *F. prausnitzii* and other *Firmicutes* correlated with improved clinical outcomes [31].

### 2.3. The Role of Machine Learning Models

Machine learning models trained on gut microbiome data have demonstrated consistent predictive performance for ICI efficacy in various solid tumors, with ongoing trials yielding promising early results [32,33].

Derosa et al. developed a gut microbiome-based predictive biomarker for ICI response using co-abundance network analysis. They identified two species-interacting groups (SIGs) associated with PD-1 blockade outcomes in NSCLC and created a topological score (TOPOSCORE) combining the SIG1/SIG2 ratio with *Akkermansia muciniphila* abundance. SIG1 taxa (e.g., *Enterocloster*, *Streptococcaceae*, *Veillonellaceae*, *and Lactobacillaceae*) correlated with shorter OS, whereas SIG2 taxa (*Faecalibacterium prausnitzii*, *Ruminococcus bicirculans*) correlated with longer OS. This biomarker showed predictive value not only in NSCLC but also in genitourinary cancers (AUC = 0.66) [34].

A multicohort prospective observational study, based on two parallel cohorts in the United Kingdom (PRIMM-UK) and the Netherlands (PRIMM-NL), investigated the gut microbiome as a biomarker of response to ICIs in advanced melanoma, collecting baseline stool samples from 165 ICI-naive patients [35]. These samples were analyzed with shotgun metagenomic sequencing and integrated with 147 publicly available samples, creating one of the largest datasets in this field. The results showed that the gut microbiome is associated with ICI response, but these associations varied significantly between cohorts, reflecting the influence of population- and treatment-specific factors. While a statistically significant difference in the gut microbiota composition of responders and non-responders was found in the UK cohort, no changes were observed in the NL one. Certain taxa, such as *Akkermansia muciniphila*, *Bifidobacterium pseudocatenulatum* and *Roseburia* spp., were enriched in responders, yet no single bacterial species consistently predicted outcomes across all datasets. Machine learning played a central role in assessing the predictive value of the microbiome. Using Lasso-based models [32,36], the authors evaluated whether microbial taxonomic and functional features could distinguish responders from nonresponders. Within individual cohorts, machine learning achieved moderate predictive performance (AUCs up to 0.78); however, microbiome prediction capability varied significantly between the two cohorts, mainly depending on the endpoint chosen (progression free survival at 12 months (PFS12) or overall response rates (ORR)). Thus, cross-cohort validation revealed limited reproducibility, with predictive accuracy dropping substantially when models trained on one cohort were applied to another. Importantly, functional gene profiles sometimes outperformed taxonomic composition, highlighting the relevance of microbial metabolic potential. Overall, the study demonstrates that the gut microbiome holds promise as a biomarker of ICI response, but its predictive power is strongly cohort-dependent.

In a meta-analysis by Limeta et al. [37], the authors analyzed baseline metagenomic data from 130 patients with metastatic melanoma in therapy with ICIs, using four previously published studies comparing responders and nonresponders to therapy. They identified microbial species enriched in responders, including *Faecalibacterium*, *Barnesiella intestinihominis* and *Ruminococcus bicirculans*, while nonresponders were enriched in taxa such as *Bacteroides thetaiotaomicron* and *Adlercreutzia equolifaciens*. Functionally, pathways linked to B vitamin metabolism were more abundant in responders, whereas nonresponders showed enrichment in aerobic respiration and nucleotide biosynthesis pathways. Machine learning was applied to integrate these microbial and functional features into a predictive model. A random forest classifier trained on the discovery cohorts achieved modest but consistent performance when tested on an independent validation set (AUC ~0.62). Among the strongest predictors were *Faecalibacterium* abundance and functional pathways related to vitamin metabolism.

A machine learning score based on multiomics (MCMLS) firstly tested on colorectal cancer was subsequently proven on datasets comprising patients with different tumors [38]. Zhou et al. analyzed three independent cohorts, Linear Discriminant Analysis (LDA) Effect Size, random forest modeling and SHAP interpretability, highlighting the role of nine genera—mainly *Akkermansia*, *Romboutsia*, *Mucispirillum* and the fungus *Rutstroemia*—as predictors of clinical response to immunotherapy in melanoma. The combined model also revealed a consistent positive interaction between *Akkermansia* and *Rutstroemia* across datasets, suggesting cross-kingdom cooperation linked to immune responsiveness [39].

Machine learning has a promising role in capturing complex microbial signatures linked to ICI outcomes, but reproducibility represents an obstacle to overcome, pointing to the need for larger, standardized and harmonized studies to develop robust microbiome-based biomarkers for clinical use.

As a final remark, gut microbiota composition has also been linked to immunotherapy-related adverse events. In a real-world study of 150 patients receiving anti-PD-1 therapy (alone or in combination), Liu et al. observed that severe adverse events correlated with enrichment in *Streptococcus*, *Paecalibacterium* and *Stenotrophomonas*, whereas *Faecalibacterium* and unidentified *Lachnospiraceae* were associated with milder toxicities. Microbial composition also varied across adverse event subtypes, including pruritus, rash, thyroid dysfunction and diarrhea [40]. Another systematic review reported that *Firmicutes* (often enriched in ICI responders) correlated with higher toxicity risk, whereas *Bacteroidetes* (frequently enriched in non-responders) were associated with a lower incidence of adverse events [41].

## 3. Gut Microbiota, HCC and ICIs

### 3.1. Gut Microbiota Composition in Patients with HCC

According to the gut–liver axis paradigm, gut dysbiosis plays a critical role in HCC pathogenesis, fostering immunosuppression and impairing cancer immunosurveillance [42]. In murine models of steatohepatitis-driven HCC, dysbiosis was associated with increased hepatic infiltration of myeloid-derived suppressor cells (MDSCs) and reduced numbers of CD4^+^ and CD8^+^ T cells. Similar patterns have been observed in human HCC, where specific taxa—particularly *Bacteroides*—were correlated with elevated circulating MDSC levels and increased expression of IL-8 and IL-13, both key mediators of MDSC recruitment and expansion [43].

**Figure 1:**
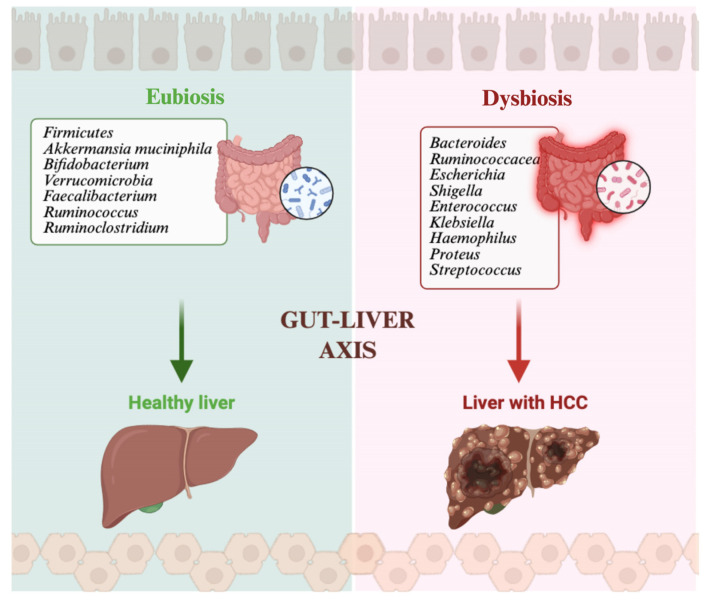
The gut–liver axis links gut microbiota composition to liver health. Eubiosis is characterized by enrichment in beneficial taxa (such as *Akkermansia muciniphila*, *Bifidobacterium*, *Faecalibacterium*, and *Ruminococcus*) that support immune homeostasis and liver integrity. Dysbiosis leads to expansion of pro-inflammatory and pathogenic species (such as *Escherichia*, *Shigella*, *Klebsiella*, and *Enterococcus*), thus promoting intestinal permeability, chronic inflammation and, ultimately, HCC development. Created in BioRender. Ianiro, G. (2026) https://BioRender.com/hqvrxz9.

Several studies have explored the gut microbiota composition in patients with liver disease, highlighting differences among healthy individuals, cirrhotic patients and those with HCC, as shown in Figure 1. In 2016, Grat et al. observed that patients with HCC reported intestinal overgrowth of *Escherichia coli* when compared to cirrhotic patients, with a sensibility of 66.7% and specificity of 73.3%, thus suggesting the potential role of gut microbiota as a useful biomarker for HCC screening in cirrhotic patients [44]. Our group demonstrated that patients with HCC and cirrhosis showed increased levels of *Bacteroides* and *Ruminococcaceae*, while *Akkermansia* and *Bifidobacterium* were reduced compared to cirrhotic patients without HCC [45]. Another study reported similar results, with HCC patients exhibiting higher levels of pro-inflammatory bacteria such as *Escherichia*, *Shigella* and *Enterococcus*, along with a significant decrease in beneficial genera like *Faecalibacterium*, *Ruminococcus* and *Ruminoclostridium* [46]. In patients with HCC, lower levels of butyrate-producing bacteria were observed, while higher levels of LPS-producing bacteria such as *Klebsiella* and *Hemophilus* spp. were found [47]. Moreover, we observed that cirrhotic patients had lower levels of protecting bacteria and showed higher intestinal inflammation (higher levels of IL18, IL13, CCL3, CCL4, CCL5 and fecal calprotectin). In the presence of HCC, an abundance of *Bacteroidetes* and a reduction in *Verrucomicrobiaceae*, *Bifidobacteriaceae*, *Akkermansia* and *Bifidobacterium* was observed [45]. The role of dysbiosis and chronic inflammation was also elucidated in a model that reported the progressive transformation from steatosis to steatohepatitis to fibrosis and finally hepatocellular carcinoma: *Bifidobacterium* and *Bacteroides* levels were inversely associated with severity of disease, with the lowest levels in HCC patients. By contrast, *Mucispirillum*, *Desulfovibrio*, *Anaerotruncus* and *Desulfovibrionaceae* consistently increased along with disease progression [48]. In another model, Clostridium difficile levels correlated with HCC development [49]. In line with these studies, Chen et al. observed that *Lactobacillus*, *Anaerostipes*, *Fusicatenibacter*, *Bifidobacterium* and *Faecalibacterium* significantly correlated with alpha-fetoprotein, transaminase and protein induced by vitamin K absence (PIVKA) levels, suggesting that these species could have an adjuvant role in the diagnosis of advanced HCC [50]. HCC tumor burden may also be influenced by gut microbiota: the presence of *Bacteroides*, *Lachnospiracea incertae sedis* and *Clostridium XIVa* is associated with poor anti-inflammatory response. Specifically, *Clostridium XIVa* is considered responsible for inhibiting NK cell responses, leading to a larger tumor burden through the conversion from primary to secondary bile acids that inhibit CXCL16 expression, which is a key regulator of NK activity [51,52].

Finally, various studies focused on the differences in gut microbiota composition in patients with HCC according to the underlying etiology of liver disease. In non-alcoholic fatty liver disease (NAFLD)-related HCC, an enrichment in *Bacteroides*, *Enterococcus* and *Ruminococcaceae* genera, coupled with a reduced abundance of *Bifidobacterium* spp., has been reported when compared with cirrhotic patients without HCC [45]. Conversely, Yan et al. found that patients with HBV-related cirrhosis and HCC displayed an increased abundance of pro-inflammatory taxa—including *Proteus*, *Klebsiella* and *Streptococcus* genera—along with a depletion of butyrate-producing bacteria (e.g., *Bacteroides phylum* and *Firmicutes* species) [53]. In another model, Ren et al. compared the fecal microbiota of 75 HBV-infected patients who developed HCC at an early stage to 105 other samples from healthy or cirrhotic patients, reporting changes in microbial diversity, with 30 specific microbial markers identified through a fivefold cross-validation on a random forest model able to differentiate among patients with and without HCC with an area under the curve of 80.64%, enlightening the role of gut microbiota as a potential biomarker for HCC detection [47].

### 3.2. Gut Microbiota Composition and ICI Efficacy in HCC

Although current evidence remains limited, the gut microbiome composition appears to influence ICI efficacy in HCC [54]. The main findings are summarized in Table 1. In a metagenomic study conducted on eight patients with HCC treated with anti-PD-1 therapy (camrelizumab) for 12 weeks, responders exhibited greater taxonomic richness and gene counts compared with non-responders. Baseline microbial composition was broadly similar between groups, with *Bacteroidetes* predominating, followed by *Firmicutes* and *Proteobacteria*. After three weeks of therapy, *Proteobacteria* significantly expanded in non-responders—dominated by *Escherichia coli* at week 12—whereas in responders, *Klebsiella pneumoniae* was the major proteobacterial member. In addition, the abundance of *Akkermansia muciniphila* and *Ruminococcaceae* from week 3 to week 12 of immunotherapy was associated with a radiological response, while the abundance of *E. coli* was a predictor of poor outcomes [55]. Mao et al. reported similar trends and identified specific taxa enriched in responders (*Lachnospiraceae bacterium-GAM79*, *Alistipes* sp. *Marseille-P5997*, *Ruminococcus callidus*, *Eubacterium siraeum*, *Gemmiger formicilis*, and *Faecalibacterium genus*) versus non-responders (predominance of *Veillonellaceae*). Reduced microbial diversity and the relative enrichment of pathogenic species were also associated with a higher incidence of immunotherapy-related adverse events, particularly colitis [56].

Zhu et al. analyzed the multikingdom gut microbiota (including gut microbiota, gut mycobiome and gut metabolites) of 80 advanced HCC patients undergoing PD-1/PD-L1 blockade, classifying them into sustained clinical benefit (SCB) and limited benefit (LB) groups according to radiological response. Distinct bacterial and metabolite profiles were observed between groups, while differences in the fungal microbiome were less pronounced. *Phascolarctobacterium faecium*, *Candidatus Avimonas narfia* and *Cladosporium* were enriched in SCB patients but undetectable in LB patients; conversely, *Actinomyces*, *Senegalimassilia_anaerobia*, *Faecalibacillus_faecis* and *Trichoderma* were significantly predominated in LB patients. The authors subsequently collected this evidence to perform a LASSO regression and develop a predictive model based on 18 bacterial species, which may predict immunotherapy response in HCC with an area under the curve value of 75.63% [57].

In a prospective cohort of 94 HCC patients treated with nivolumab or pembrolizumab, Lee et al. reported that disease progression was associated with the expansion of *Prevotellaceae* and *Enterobacteriaceae*, alongside a reduction in *Lachnospiraceae* and *Veillonellaceae*—the latter being more abundant in responders. Improved progression-free survival (PFS) and overall survival (OS) correlated with a favorable microbial signature, characterized by depleted *Prevotella 9* and enriched *Lachnoclostridium*. Fecal metabolomic profiling revealed significantly higher levels of secondary bile acids—including ursodeoxycholic acid (UDCA), tauro-UDCA, ursocholic acid (UCA) and murideoxycholic acid (MDCA)—in responders, with positive correlations with *Lachnoclostridium* abundance [58].

Chung et al. conducted a study on a small cohort of eight advanced HCC patients receiving nivolumab and classified according to radiological response assessed by the RECIST 1.1 criteria. They reported that responders had a higher Shannon diversity index and greater beta-diversity compared with non-responders. Alterations in the *Firmicutes/Bacteroidetes* and *Prevotella/Bacteroides* ratios were observed in non-responders, whereas *Akkermansia* was uniquely present in responders. Specific taxa—including *Dialister pneumosintes*, *Escherichia coli*, *Lactobacillus reuteri*, *Streptococcus mutans*, *Enterococcus faecium*, *Streptococcus gordonii*, *Veillonella atypica*, *Granulicatella* spp. and *Trichuris trichiura*—were enriched in non-responders, while *Citrobacter freundii*, *Azospirillum* spp. and *Enterococcus durans* predominated in responders [59]. These findings have been confirmed in several studies and suggest a potential role of the gut microbiome in predicting response to immunotherapy in patients with HCC. For example, Ponziani et al. found that relative abundance of Akkermansia was increased, whereas that of Enterobacteriaceae was reduced, in HCC patients treated with Tremelimumab and/or Durvalumab, who achieved disease control [60]; Zheng et al. similarly found that *Akkermansia muciniphila* and *Ruminococcaceae* spp. were enriched in responders, whereas *Proteobacteria* increased and became predominant by week 12 of ICI treatment in non responders [55]. Wu et al. also demonstrated that the abundance of *Akkermansia muciniphila* was remarkably reduced in patients with advanced HCC and its levels inversely correlated with the response to ICIs [61]. Finally, Lee et al. conducted a study on microbiota signatures in patients with viral and non-viral HCC receiving first-line immunotherapy. Fecal *Bateroides ovatus*, *Kluyvera georgiana*, *Klebsiella oxytoca* and *Enterococcus faecium* were predominant in MASLD-HCC, while viral HCC was associated with a higher abundance of *Bifidobacterium*. Durable response in MASLD HCC was observed in patients with fecal samples enriched with *Mediterraneibacter gnavus ATCC 29149* and a higher concentration of short-chain fatty acids and UDCA [62].

## 4. Influence of Gut Microbiota Metabolites on ICI Efficacy in HCC

Taxonomic composition has been the most extensively investigated dimension of the microbiome; however, increasing evidence suggests that its functional output, particularly metabolite production, may represent an even closer mediator of ICI efficacy, although the mechanisms have yet to be fully understood. As far as we know, multiple mechanisms are involved, including the gut–liver axis, anti-tumor immune response and tumor microenvironment modulation, all interconnected [63]. Several association studies have highlighted the correlation between the levels of certain metabolites and the treatment response. Among the main actors, we include short-chain fatty acids (SCFAs), bile acids (BAs), inosine, tryptophan and its catabolites and Trimethylamine-N-oxide (TMAO). All of them can be measured non-invasively and easily in feces, making them excellent candidates as possible future biomarkers.

### 4.1. Short-Chain Fatty Acids

SCFAs are small fatty acids containing fewer than six carbon atoms, produced by specific gut bacterial groups through the fermentation of non-digestible or low-digestible carbohydrates (e.g., dietary fibers, inulin, polysaccharides and oligosaccharides). The three principal SCFAs—acetate, propionate and butyrate—are generated mainly by members of the Lachnospiraceae and Ruminococcaceae families, which express butyrate kinase and butyrate CoA-transferase, two key enzymes in SCFA biosynthesis [64,65].

SCFAs exert multiple beneficial effects on gut health and immune function, fostering a favorable tumor microenvironment that counteracts carcinogenesis. They reduce intestinal permeability and thereby limit the translocation of lipopolysaccharides (LPS) and their pro-inflammatory effects in peripheral tissues. In particular, butyrate enhances the expression of the tight junction (TJ) protein claudin-1 and promotes the redistribution of occludin and zonula occludens-1 (ZO-1) to the cellular membrane. In parallel, SCFAs stimulate mucin production, strengthening the intestinal barrier [66]. SCFAs also contribute to intestinal homeostasis by influencing regulatory T-cell (Treg) proliferation and interleukin production. As key mediators of the gut–liver axis, they directly and indirectly regulate hepatic homeostasis and physiological liver function. SCFAs stimulate glucagon-like peptide-1 (GLP-1) secretion from enteroendocrine cells, ultimately improving insulin sensitivity. This occurs via activation of the G-protein-coupled receptors GPR41 and GPR43 on hepatocytes, enhancing glucose uptake and glycogen synthesis [67]. In a mouse model of non-alcoholic fatty liver disease (NAFLD), Zhou et al. demonstrated that butyrate reversed GLP-1 receptor downregulation in hepatic cells, leading to insulin receptor upregulation [68]. Furthermore, SCFA–GPR41/GPR43 signaling promotes antimicrobial peptide expression. For example, propionate binding to GPR43 on liver cancer cells has been shown to inhibit their growth, while butyrate reduces tumor necrosis factor-alpha (TNF-α) production by Kupffer cells and increases prostaglandin E2 (PGE2) expression, exerting an anti-inflammatory effect [69,70,71].

SCFAs also modulate adaptive immunity by influencing CD4^+^ and CD8^+^ T cells. Butyric acid (BA) induces FOXP3^+^ CD4^+^ Treg differentiation and, together with other SCFAs, promotes CD8^+^ T-cell IL-17 production as well as IFN-γ and granzyme B expression. Both BA and valeric acid (VA) act as histone deacetylase (HDAC) inhibitors [72,73,74]. HDAC inhibition has been associated with upregulation of PD-1 ligands and suppression of apoptosis in intratumoral CD4^+^ T cells, thereby enhancing immunotherapy responses in melanoma models [75,76]. In pancreatic cancer models, SCFAs similarly enhance anti-tumor reactivity by reinforcing CD8^+^ T-cell effector functions [77].

SCFAs have been consistently implicated in modulating the response to ICI therapy in solid tumors. In a prospective cohort of 52 patients with various solid malignancies receiving PD-1 inhibitors (nivolumab or pembrolizumab), elevated concentrations of SCFAs—including fecal acetic acid, propionic acid, butyric acid, valeric acid and plasma isovaleric acid—were associated with significantly longer progression-free survival [78]. A recent prospective study observed that in patients with metabolic dysfunction-associated steatotic liver disease-related HCC and viral hepatitis-related HCC treated with first-line combination immunotherapy, a higher fecal acetate level was a predictor of better prognosis with longer overall survival, progression-free survival and durable response [62]. Mechanistic insights have also been provided in preclinical studies. Hu et al. demonstrated that, in vitro, acetate suppresses interleukin-17A (IL-17A) production by liver-derived type 3 innate lymphoid cells (ILC3s) in a dose-dependent manner via downregulation of Sox13 mRNA. Given that ILC3 infiltration correlates with worse outcomes in HCC, the authors evaluated the effects of SCFAs in murine HCC models. Mice receiving acetate-enriched drinking water—either alone or in combination with PD-1 therapy—exhibited reduced PD-1 expression on ILC3s and decreased IL-17A release, particularly in the combination group [79].

Alterations in SCFA levels have also been reported in HCC compared with healthy controls. In murine models, SCFA concentrations—particularly acetate—were reduced, accompanied by a decline in Lactobacillus reuteri abundance [46]. In early-stage human HCC, both the abundance of butyrate-producing genera and fecal butyrate levels were diminished, likely due to enhanced butyrate metabolism [47,80].

**Figure 2:**
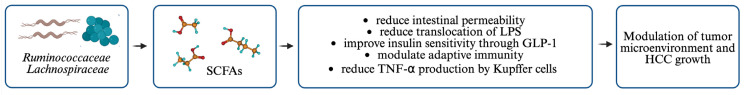
Schematic representation of short-chain fatty acids’ (SCFAs) role in HCC during treatment with ICIs. LPS: lipopolysaccharide; GLP-1: glucagon-like peptide 1; TNF: tumor necrosis factor; HCC: hepatocellular carcinoma. Created in BioRender. Ianiro, G. (2026) https://BioRender.com/hqvrxz9.

Collectively, these findings suggest that SCFAs contribute to anti-HCC activity by improving the tumor microenvironment and augmenting the efficacy of ICIs. Given their stability, the possibility of non-invasive fecal measurement and their correlation with treatment outcomes, SCFAs represent promising prognostic biomarkers in HCC (Figure 2).

### 4.2. Bile Acids

**Figure 3:**
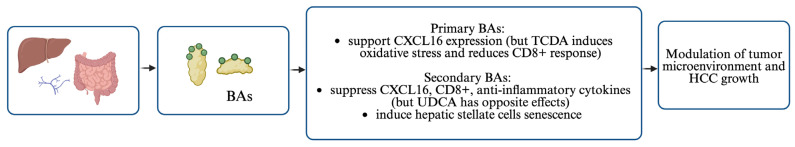
Schematic representation of bile acids’ (BAs) role in HCC during treatment with ICIs. TCDA: taurochenodeoxycholic acid; UDCA: ursodeoxycholic acid; HCC: hepatocellular carcinoma. Created in BioRender. Ianiro, G. (2026) https://BioRender.com/hqvrxz9.

Bile acids are synthesized in the liver via cholesterol hydroxylation, yielding primary bile acids—cholic acid (CA) and chenodeoxycholic acid (CDCA)—which are conjugated with taurine or glycine and secreted into the duodenum. In the gut, bacterial enzymes convert them into secondary bile acids, primarily deoxycholic acid (DCA) from CA and lithocholic acid (LCA) from CDCA [81]. Secondary BAs are reabsorbed through the portal circulation, with a fraction entering the systemic circulation. In the intestinal lumen, they exert antimicrobial effects by disrupting bacterial membranes and inducing antimicrobial peptide production [82]. Within hepatic sinusoidal endothelial cells (LSECs), secondary BAs suppress the expression of the chemokine CXCL16, which recruits natural killer T (NKT) cells to the liver to target HCC cells and metastases. This suppression fosters a tumor-permissive microenvironment. Conversely, primary BAs stimulate CXCL16 expression and exert opposing effects [83]. For instance, LCA reduces hepatic CXCL16 levels, intra-tumoral NK T cells, CD8^+^ T cells, IFN-γ and TNF production, thereby promoting HCC growth, whereas DCA induces hepatic stellate cell senescence, generating a pro-inflammatory, pro-tumor milieu [84,85]. However, not all primary BAs are protective. Varanasi et al. demonstrated that taurochenodeoxycholic acid (TCDA) accumulates in the tumor microenvironment, induces oxidative stress and reduces CD8^+^ T-cell infiltration in a dose-dependent fashion, ultimately facilitating tumor progression and impairing ICI efficacy; therefore, the inhibition of bile acid synthesis in hepatocytes through deletion of the conjugating enzyme BAAT positively sensitized tumors to anti-PD-1 therapy in experimental models [86]. In contrast, the secondary BA ursodeoxycholic acid (UDCA) enhances CD4^+^ and CD8^+^ T-cell responsiveness by suppressing TGF-β expression and promoting granzyme B release [85,87]. Clinical observations have linked higher fecal levels of various secondary BAs, including UDCA, with favorable ICI responses in HCC patients, correlating with increased *Lachnoclostridium* abundance, whereas elevated LCA levels have been associated with disease progression [58,88]. A recent study demonstrated higher UDCA levels in durable responders between patients with MASLD-related HCC and viral-related HCC treated with first-line combination immunotherapy [62]. All this evidence suggests that modifying BA synthesis or dietary supplementation with certain BAs could improve HCC response to ICI therapy. Although the specific roles of individual BAs in HCC remain incompletely understood, disruptions in primary–secondary BA homeostasis clearly influence tumor development and therapeutic responsiveness. Notably, FXR-knockout mice exhibit a 20% higher incidence of HCC, whereas FXR activation by BAs mitigates tumor formation [84]. Similar to SCFAs, BAs can be quantified non-invasively in fecal samples, making them attractive candidates as prognostic biomarkers for HCC (Figure 3).

### 4.3. Inosine

Inosine is a purine nucleoside derived from extracellular purine adenosine by the enzymatic action of adenosine deaminase (ADA). Adenosine has a very short half-life before its conversion to inosine and is itself derived from ATP, thanks to the enzymes CD39 and CD73 ectonucleotidases [89].

Several gut bacteria utilize purines as an energy source under the anaerobic conditions typical of the intestine. Microbial purine degraders are important modulators of host purine homeostasis in the gut and in circulation. Mager et al. identified Bifidobacterium pseudolongum as the main inosine producer, mainly in the duodenum [90,91].

Some preclinical studies have shown that inosine is involved in enhancing the anti-tumor response and amplifying the efficacy of ICI therapy in mouse models of melanoma and colorectal cancer. Tanoue et al. demonstrated that cecal and serum levels of inosine were increased in germ-free (GF) mice inoculated with an 11-member bacterial consortium compared to other GF mice, and that inoculated GF mice had higher levels of INF-γ positive CD8^+^ T cells with a strong inhibition of tumor growth in conjunction with ICIs [92]. Mager et al. demonstrated that Bifidobacterium pseudolongum-monocolonized GF mice had the highest abundance of inosine and the best response to ICI therapy: decreased gut barrier function induced by immunotherapy increased systemic translocation of inosine and activated anti-tumor T cells (CD4^+^ and CD8^+^ T-cells, thereby increasing IFN-γ release); the effect of inosine required co-stimulation and was dependent on the adenosine A2A receptor expressed on T cells [90]. Basically, inosine directs differentiation of Th1 cells in an adenosine receptor A2A-dependent manner, overcoming tumor cell-intrinsic resistance to immunotherapy [90]. Furthermore, inosine supports T-cell proliferation and differentiation, being an alternative carbon source for CD8^+^ T cells in glucose restriction conditions, as in TME [93]. In addition, inosine directly inhibits UBA6 (ubiquitin-like modifier activating enzyme 6): through mouse melanoma and breast cancer models, it emerged that genetic ablation of UBA6 led to higher ICI sensitivity than wild-type models [94].

**Figure 4:**
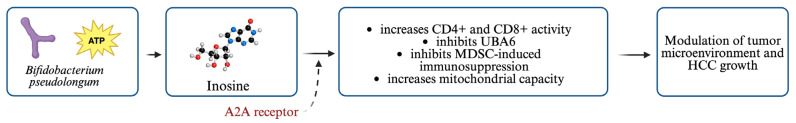
Schematic representation of inosine’s role in HCC during treatment with ICIs. ATP: adenosine triphosphate; UBA6 ubiquitin-like modifier activating enzyme 6; MDSC: myeloid-derived suppressor cells; HCC: hepatocellular carcinoma. Created in BioRender. Ianiro, G. (2026) https://BioRender.com/hqvrxz9.

In a randomized, controlled phase 2 study, 172 patients with advanced solid tumors, including HCC, were randomly assigned to receive ICI therapy (PD-1/PD-L1 inhibitors) plus inosine or ICI therapy without inosine; in the inosine group, both a 2.6-month increase in PFS and a 37% reduction in the risk of disease progression were observed compared to the non-inosine group. Furthermore, the tendency for inosine to reduce the adverse effects of immunotherapy was noted [95]. The ability of inosine to enhance CD8^+^ T-cell function and inhibit MDSC-induced immunosuppression is highly relevant in HCC [95]. In a mouse model of HCC treated with engineered chimeric antigen receptor T (CAR-T), CAR-T cell migration and resistance to TGF-β1 inhibition is favored by the adenosine–inosine conversion [89]. Finally, it has been shown that inosine generates a series of metabolic alterations that lead to reduced glycolysis and increased mitochondrial capacity and that it increases the expression of the adenosine A2A receptor after treatment with ICI. These changes may persist and positively influence the long-term response to ICI (Figure 4) [89,96].

### 4.4. Tryptophan Catabolites

Tryptophan is an essential amino acid processed by gut microbiota into various active metabolites. Tryptophanase is expressed by many Gram-negative and Gram-positive bacterial species including *Escherichia coli*, *Clostridium* spp. and *Bacteroides* spp., leading to indole production. Indoleamine 2,3-dioxygenase 1 (IDO1), IDO2 and tryptophan 2,3-dioxygenase 2 (TDO2) are key enzymes involved in the degradation of tryptophan into kynurenine (Kyn); other catabolites of tryptophan include tryptamine, indoleethanol (IE), indolepropionic acid (IPA), indolelactic acid (ILA), indoleacetic acid (IAA), skatole, indolealdehyde (IAld) and indoleacrylic acid (IA) [97,98].

Tryptophan catabolites exert multiple actions; namely, they regulate intestinal barrier function through the aryl hydrocarbon receptor (AhR) and pregnane X receptor (PXR) and modulate the secretion of glucagon-like peptide-1 (GLP-1), with less insulin resistance, a lower fasting blood glucose level and reduced appetite as a consequence [98]. Through AhR indole and kynurenine, they regulate tumor-associated macrophage (TAM) polarization and T-cell function, inducing an immunosuppressive tumor microenvironment. In a model of pancreatic ductal adenocarcinoma, Hezaveh et al. identified AhR responsible for Arg1 and IL10 expression in TAMs and IFNγ expression inhibition in CD8^+^ T cells; they found a correlation between AhR expression and poor outcomes in humans [97,99]. In a preclinical study, it was demonstrated that TDO2 activates the kynurenine–AhR pathway and promotes the epithelial to mesenchymal transition, participating in the process of metastatic invasion of HCC. The inhibition of TDO2 expression leads to lower kynurenine levels and inactivates the AhR, resulting in the activation and proliferation of CD3^+^ T cells and in the promotion of an anti-tumor microenvironment [100,101].

**Figure 5:**
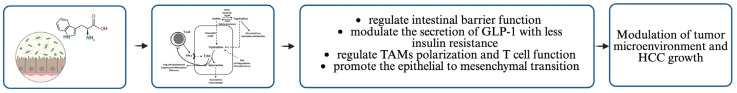
Schematic representation of tryptophan catabolites’ role in HCC during treatment with ICIs. GLP-1: glucagon-like peptide 1; TAMs: tumor-associated macrophages; HCC: hepatocellular carcinoma; ICIs: immune-checkpoint inhibitors Created in BioRender. Ianiro, G. (2026) https://BioRender.com/hqvrxz9.

In HCC patients the gut microbiota composition is remarkably altered with a decrease of Firmicutes populations, particularly Blautia species, which possess tryptophan-metabolizing capacity and with a rise in Proteobacteria, which leads to reduced indole-3-acetic acid production [102]. In HCC TME tryptophan metabolism is critically dysregulated with an overexpression of IDO1, IDO2 and TDO2. This enhances kynurenine and indole pathways with the depletion of tryptophan in the liver, which leads to an immunotolerant TME, reducing the proliferation of effector T lymphocytes and favoring the differentiation of regulatory T (Treg) cells [103,104,105]. Anti-CTLA-4 and anti-PD-1 therapies promote the upregulation of IDO1 in resistant HCC tumors. Therefore, a negative feedback mechanism is generated, whereby the immune activation induced by ICI therapy leads to tryptophan catabolism with the immunosuppressive effects described above [106]. This was confirmed by early phase clinical trials, as targeting IDO1/TDO and their downstream effectors in combination with ICI has shown promising results [107]. After ICI therapy, patients with sustained response showed maintenance of a restored tryptophan metabolism compared to non-responders. Basically, the kynurenine pathway influences TME, promoting the development of a tumor immune-tolerant environment in either enzymatic or non-enzymatic manners. Bekki et al. demonstrated that in chronic HCV patients, kynurenine production gradually increased when chronic HCV progressed to HCC and a high level of serum kynurenine correlated with poor prognosis of HCC [108,109]. Tryptophan metabolites represent promising prognostic and response biomarkers for ICI therapy. In NSCLC plasma concentration of 3-hydroxyanthranilic acid (3-HAA), a downstream kynurenine metabolite known to have anti-inflammatory activity has already been identified as a possible biomarker for longer progression-free survival in response to ICI therapy [110]. Assessment of plasma tryptophan metabolites is a non-invasive and repeatable method which can also be applied promisingly in HCC (Figure 5).

### 4.5. Trimethylamine-N-Oxide

**Figure 6:**
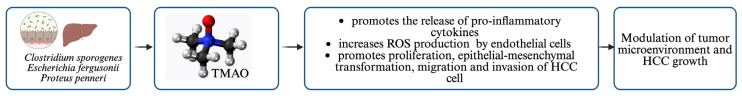
Schematic representation of Trimethylamine-N-oxide’s (TMAO) role in HCC during treatment with ICIs. ROS: reactive oxygen species; HCC: hepatocellular carcinoma. Created in BioRender. Ianiro, G. (2026) https://BioRender.com/hqvrxz9.

TMAO derives from dietary choline, L-carnitine and betaine through gut bacteria metabolism. *Clostridium sporogenes*, *Escherichia fergusonii* and *Proteus penneri* are first involved in producing trimethylamine (TMA), which is then transported to the liver and converted into TMAO by the flavin monooxygenase (FMO) family, in particular FMO3 [111,112]. TMAO has a pro-inflammatory effect, promoting the release of pro-inflammatory cytokines and the production of reactive oxygen species (ROS) by endothelial cells. Therefore, it is well known that TMAO plays a significant role in atherogenesis and in increasing cardiovascular risk [113]. Recently, some studies have demonstrated that in HCC, TMAO promotes proliferation, epithelial–mesenchymal transformation, migration and the invasion of HCC cells thanks to MAPK pathway activation and periostin (POSTN) upregulation in the presence of TNF-α; high expression of POSTN was associated with neutrophil infiltration. Accordingly, Liu et al. observed that higher serum levels of TMAO were associated with a higher risk of developing primary liver tumors, above all in patients with simultaneous lower serum levels of choline [114,115]. Therefore, pre-treatment TMAO concentrations may serve as a biomarker for poor immunotherapy response; however, genetic polymorphisms for FMO3 and the influence of dietary intake of precursors for production of TMAO may make the assessment of a TMAO level cut-off harder for poor or good prognosis (Figure 6).

**Figure 7:**
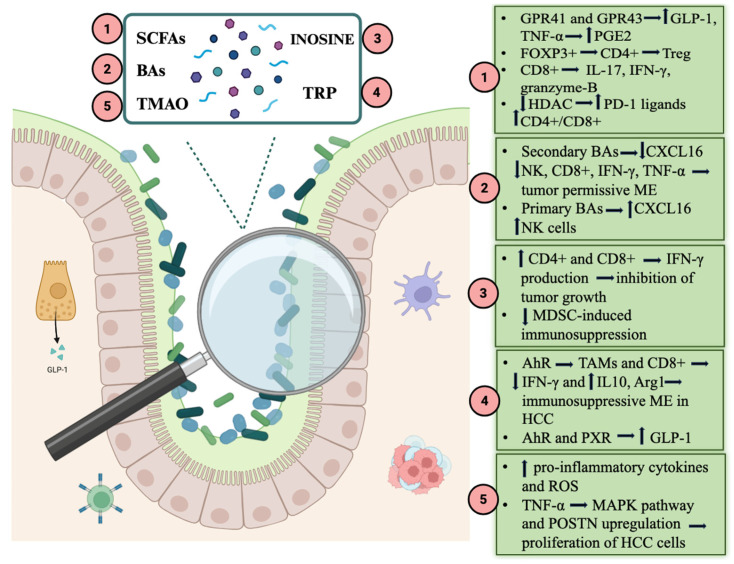
Schematic summary representation of gut microbiota-derived metabolites influencing the tumor microenvironment and response to ICIs in HCC. Short-chain fatty acids (SCFAs), bile acids (BAs), inosine and tryptophan (TRP) catabolites modulate immune signaling through G-protein-coupled receptors (GPR41/43), histone deacetylase (HDAC) inhibition, aryl hydrocarbon receptor (AhR) and pregnane X receptor (PXR) pathways, enhancing glucagon-like peptide-1 (GLP-1) secretion and shaping T-cell responses. Primary BAs increase CXCL16 and natural killer (NK) cell recruitment, whereas secondary BAs promote a tumor-permissive microenvironment. SCFAs and inosine enhance CD4^+^ and CD8^+^ T-cell activity and interferon-γ (IFN-γ) production, while tryptophan metabolites contribute to immunosuppression via tumor-associated macrophages (TAMs). Trimethylamine-N-oxide (TMAO) promotes inflammation and HCC proliferation through reactive oxygen species (ROS) and mitogen-activated protein kinase (MAPK) signaling. ME: microenvironment; MDSC: myeloid-derived suppressor cells; HCC: hepatocellular carcinoma; GLP-1: glucagon-like peptide 1; POSTN: periostin. Created in BioRender. Ianiro, G. (2026) https://BioRender.com/hqvrxz9.

Figure 7 summarizes the main gut microbiota metabolites and the pathways through which they influence response to ICIs in HCC patients.

## 5. Future Perspectives: Microbiota Modulation as a Therapeutic Strategy

As previously discussed, intestinal eubiosis may enhance the therapeutic efficacy of ICIs. Accordingly, strategies such as fecal microbiota transplantation (FMT), live biotherapeutic products, defined bacterial consortia, probiotics, prebiotics, synbiotics and dietary modulation represent promising approaches to improving ICI response rates [116,117].

FMT, defined as the transfer of gut microbiota from a healthy donor into the gastrointestinal tract of a recipient, has been the focus of several phase I/II trials in malignancies such as melanoma, non-small-cell lung cancer, epithelial tumors, renal cell carcinoma and colorectal cancer, with encouraging results [117]. In a study of 10 anti-PD-1-refractory patients, Baruch et al. reported clinical responses in three patients (two partial, one complete) following FMT. In another trial, 6 of 15 refractory patients achieved partial or complete responses or disease stabilization. In these responders, the gut microbiome shifted toward the donor profile, with increased CD8^+^ T-cell activation, reduced IL-8-producing myeloid cells, enrichment of favorable taxa (*Lachnospiraceae*, *Ruminococcaceae* and *Bifidobacteriaceae*) and depletion of unfavorable species such as *Bacteroides* [118,119]. In a seminal preclinical study, Routy et al. demonstrated that FMT from ICI-responder cancer patients into germ-free or antibiotic-treated mice promoted CXCR3^+^ CD4^+^ T-cell proliferation within the tumor microenvironment and upregulated PD-L1 expression on splenic T cells, thereby potentiating the anti-tumor effects of PD-1 blockade. In contrast, FMT from non-responder donors failed to elicit such effects [120].

Evidence also supports a role of microbiota modulation in HCC. In a murine HCC model, Lactobacillus reuteri levels were markedly reduced in fecal samples. To further explore causality, Hu et al. transplanted feces from wild-type mice, *L. reuteri*, *L. reuteri* + antibiotics and HCC-bearing mice into recipient HCC mice. Although all developed typical HCC with fibrosis, those receiving wild-type or *L. reuteri* stools exhibited fewer and smaller liver tumors, along with significantly lower markers of hepatic injury (AST, ALT, ALP, γGT) [79].

A possible alternative to FMT is MET40, a defined microbial ecosystem comprising 30 cultured intestinal bacterial strains from a cancer-free donor. In a recent monocenter trial in ICI-naïve patients with advanced solid tumors, MET40 was well tolerated and induced a microbiota shift towards taxa associated with favorable responses (*Enterococcus*, *Bifidobacterium*, and *Phascolarctobacterium*) without increasing the incidence of severe adverse events [121].

Safety remains an important consideration, yet current evidence supports the feasibility of these interventions [27]. Moreover, growing data suggest an association between baseline microbiome composition and ICI-related toxicities, highlighting the potential of microbiota modulation (including FMT) not only to enhance efficacy but also to mitigate adverse events [122,123].

## 6. Discussion

ICIs have revolutionized the field of oncology in the treatment of numerous solid tumors, with evidence of promising results. However, approximately 60% of patients with HCC do not respond adequately to ICI therapy [7]. Therefore, understanding the mechanisms determining this resistance to treatment and identifying reliable and easy-to-use biomarkers of response or non-response to therapy is critical. The ultimate goal is to stratify patients according to therapeutic outcomes and intervene to increase response where possible.

Several predictive biomarkers have already been studied and partially approved as indicators of response to ICI therapy in solid cancers, such as DNA damage response (DDR) gene alterations, MHC genotypes, beta-2-microglobulin (B2M) deficiency, polymerase epsilon (POLE) mutations, Janus kinase 1/2 (JAK1/2) mutations, tumor-infiltrating T cells (TILs) and peripheral T cells [124]. The gut microbiome and its metabolites represent a promising non-invasive biomarker, which has gained an increasingly important role in more recent studies on immunotherapy and cancer. In this regard, the MITRE trial is a large-scale multicentric study which will analyze and elaborate a gut microbiome signature which could predict ICI efficacy in patients with advanced cancers [15]. The composition and functional activity of the gut microbiota appear to influence treatment outcomes, reflecting the complex interplay between microbial ecology, systemic inflammation and immune modulation. Studies have shown that responders to ICIs often display higher microbial diversity, enrichment in beneficial taxa such as *Akkermansia muciniphila*, *Faecalibacterium prausnitzii* and members of the *Ruminococcaceae* and *Lachnospiraceae* families, together with a relative depletion of potentially pathogenic Proteobacteria and Enterobacteriaceae. These microbial signatures are consistently associated with improved radiological response, PFS and OS. Conversely, non-responders typically exhibit reduced butyrate-producing bacteria and expansion of pro-inflammatory species which also correlate with immunotherapy-related adverse events [56].

Increasing interest is being directed towards the role of gut microbiota metabolites, critically capable of modulating the tumor microenvironment and response to ICIs. Responders show increased synthesis of SCFAs which correlate with prolonged survival and exert direct immunomodulatory effects by promoting epithelial integrity, reducing pro-inflammatory cytokines and enhancing CD8^+^ T-cell cytotoxicity. Similarly, secondary BAs such as UDCA correlate with ICI responsiveness, whereas primary BAs (DCA or LCA) are linked to disease progression. Other metabolites such as inosine, derived from *Bifidobacterium* species, have been shown to potentiate anti-PD-1 efficacy by activating A2A receptors on effector T cells, while an increase in kynurenine, derived from tryptophan degradation, reflects an immunosuppressive metabolic milieu mediated by IDO1 and TDO2 activity. These data suggest that both microbial composition and metabolic function contribute to shaping the immune response to ICIs.

In this regard, machine-learning approaches have further clarified the predictive potential of microbiota-derived biomarkers. Algorithms applied to metagenomic and metabolomic datasets have identified reproducible microbial signatures associated with clinical benefit, often highlighting *Faecalibacterium* abundance and vitamin B or SCFA biosynthetic pathways among the most informative variables. While these models achieve moderate accuracy within single cohorts, their performance decreases when applied across different populations, reflecting the strong impact of geographic, dietary and methodological heterogeneity. Nevertheless, their use underscores how artificial intelligence can integrate complex biological layers—taxonomic, functional and metabolic—into composite indicators of therapeutic response.

The microbiota also appears to influence treatment-related toxicity. This dual role opens the possibility of using microbial signatures not only to predict response but also to stratify the risk of adverse immune reactions, paving the way for a more personalized therapeutic approach [40].

Interventional strategies targeting the gut microbiota are now moving from preclinical evidence to clinical application. FMT from ICI responders has been shown to enhance antitumor immunity in refractory patients and to restore sensitivity to checkpoint blockade. Similarly, administration of specific probiotics or next-generation bacterial consortia has demonstrated the ability to enrich beneficial taxa, modulate cytokine profiles and improve immune activation without increasing toxicity. These findings collectively support the development of microbiota-centered adjuvant strategies to complement immunotherapy in HCC [116].

However, methodological heterogeneity strongly impacts the quality of evidence on this topic. Despite the growing interest in microbiota-based biomarkers, the microbial signatures associated with ICI response in HCC remain only partially concordant across studies. For example, *Akkermansia muciniphila*, *Faecalibacterium* and *Ruminococcaceae* were associated with benefit in some cohorts, whereas other studies identified *Veillonellaceae*, *Lachnoclostridium*, *Phascolarctobacterium faecium* or even distinct etiology-specific taxa as markers of response. Likewise, taxa linked to poor outcomes were not fully overlapping, including *Escherichia coli*, *Prevotellaceae*, *Enterobacteriaceae*, *Veillonellaceae* or *Actinomyces* in different series. These discrepancies may reflect the differences in geography, underlying liver disease etiology, dietary background, and exposure to antibiotics or other concomitant drugs among the cohorts. In addition, there is still no standardization of methods of sample collection and analysis and studies differed substantially in sample size, timing of stool collection, sequencing strategy, and bioinformatic processing, all of which may affect taxonomic and functional outputs. Therefore, current contradictions may be the expression of highly context-dependent and still insufficiently standardized microbiome–ICI associations.

Furthermore, from a clinical perspective, the translation of microbiome biomarkers into routine practice remains challenging. First, cohort variability is substantial across available studies, not only in terms of geography and diet, but also with regard to HCC etiology, disease stage, liver function, prior antibiotic exposure, and type of immunotherapy administered, all of which may influence microbiome composition and treatment outcomes. Standardization is another major unmet need, since differences in each of these steps can generate non-comparable results across studies: stool collection, storage, sequencing platforms, taxonomic resolution, and bioinformatic processing. In addition, most currently proposed signatures derive from relatively small, single-cohort experiences and show only moderate reproducibility when externally applied, thus limiting immediate clinical utility.

Finally, for implementation in clinical decisions, microbiome biomarkers would also require regulatory validation. Demonstrations of analytical robustness, clinical validity, added value over existing biomarkers, clear frameworks for quality control, data interpretation, and integration into diagnostic workflows are needed. In this regard, prospective multicenter studies with harmonized and standardized methodologies will be essential to move these biomarkers from exploratory research to real-world clinical application [109].

In conclusion, looking ahead, future research should aim to standardize microbiome sampling and analytical methods in prospective multicenter studies, stratified by etiology and treatment regimen. Integrating metagenomics, metatranscriptomics and metabolomics with tumor genomic and immunologic data will help build more accurate and generalizable predictive models and clarify causal mechanisms linking microbial metabolites to immune modulation. Moreover, gut microbiota modulation strategies such as diet, prebiotic supplementation and targeted metabolite delivery could offer innovative, non-invasive ways to enhance immunotherapy efficacy. Finally, combining microbiome-derived efficacy and toxicity signatures into clinical decision algorithms may represent a decisive step toward a truly personalized management of HCC in the era of immunotherapy.

## Figures and Tables

**Table 1:** Summary of gut microbial taxa associated with response and non-response to immune checkpoint inhibitors in patients with hepatocellular carcinoma. MASLD: metabolic dysfunction-associated steatotic liver disease; HCC: hepatocellular carcinoma.

Study	Taxa Associated with Response/ Sustained Clinical Benefit	Taxa Associated with Non-Response/Limited Benefit/Progression
Zheng et al. [51]	*Akkermansia muciniphila*; *Klebsiella pneumonia*; *Ruminococcaceae*	*Escherichia coli*
Mao et al. [52]	*Lachnospiraceae* bacterium-GAM79; *Alistipes* sp.; *Marseille-P5997*; *Ruminococcus callidus*; *Eubacterium siraeum*; *Gemmiger formicilis*; *Faecalibacterium genus*	*Veillonellaceae*
Zhu et al. [53]	*Phascolarctobacterium faecium*; *Candidatus Avimonas narfia*; *Cladosporium*	*Actinomyces*; *Senegalimassilia anaerobia*; *Faecalibacillus faecis*; *Trichoderma*
Lee et al. [54]	Veillonellaceae; *Lachnoclostridium*	Prevotellaceae; Enterobacteriaceae; Prevotella 9
Chung et al. [55]	*Akkermansia*; *Citrobacter freundii*; *Azospirillum* spp.; *Enterococcus durans*	*Dialister pneumosintes*; *Escherichia coli*; *Lactobacillus reuteri*; *Streptococcus mutans*; *Enterococcus faecium*; *Streptococcus gordonii*; *Veillonella atypica*; *Granulicatella* spp.
Li et al. [57]	*Faecalibacterium*	Bacteroidales
Wu et al. [58]	Higher abundance of *Akkermansia muciniphila*	Reduced abundance of *Akkermansia muciniphila*
Lee et al. [59] (etiology-stratified cohort)	In MASLD-HCC: *Mediterraneibacter gnavus* ATCC 29149	Not specifically reported in the text

## Data Availability

No new data were created or analyzed in this study. Data sharing is not applicable to this article.
